# Benefits of surf therapy for children with disabilities in South Africa: a single case story

**DOI:** 10.3389/fspor.2025.1703720

**Published:** 2026-01-22

**Authors:** Roxanne Davis, Yumna Albertus, Angus Hunter, Theresa Lorenzo

**Affiliations:** 1 Department of Health and Rehabilitation Science, Division of Disability Studies, Faculty of Health Sciences, University of Cape Town, Cape Town, South Africa; 2 Health Through Physical Activity, Lifestyle and Sport, Faculty of Health Sciences, University of Cape Town, Cape Town, South Africa; 3 Nottingham Trent University, Nottingham, United Kingdom

**Keywords:** bioecological systems theory, children with disabilities, community based rehabilitation, disability, surf therapy, surfing

## Abstract

**Introduction:**

There is a well-established association between physical activity and positive health and wellbeing outcomes, however, evidence on the effectiveness of surf therapy as a therapeutic intervention for children with disabilities remains limited. This article presents a longitudinal case narrative of Rowan, one of five children who participated in a structured surf therapy programme in the Western Cape province of South Africa as part of a broader PhD study involving 35 participants across multiple stakeholder groups. This case was purposefully selected to highlight in-depth individual experience over time.

**Method:**

A qualitative participatory research approach was used to explore the benefits of a surf therapy programme from the perspective of children. The research design was a longitudinal exploratory case study underpinned by interpretive phenomenological analysis of themes and sub-themes. Data were gathered through three open-ended narrative interviews with each child over a one year period.

**Findings and discussion:**

The key theme: “An ambassador's journey of change” describes the child's experience of and perspectives on the benefits of surf therapy under three sub-themes: “Surfing has taught me to be more myself”; “Re-shaping my worldview” and “Now I like to be (am) stress free”. The mental, physical, social, and emotional health benefits of surf therapy for one child at micro, meso, exo and macro systems levels are discussed, highlighting the relevance of Social-Ecological Model of Disability and the Community-Based Rehabilitation Guidelines of the World Health Organisation to facilitate disability inclusion.

**Conclusion:**

The case story of Rowan illustrates how an inclusive surf therapy programme supports the health, development, and relational wellbeing of children with disabilities by reshaping their worldview and strengthening self-confidence. The programme enabled the mastery of new skills that extended beyond the ocean into their homes, schools, and broader ecological systems. This study advances surf therapy research by offering rare longitudinal evidence from a Global South context. It demonstrated how multisystem mechanisms of change, rooted in disability inclusive and community-based frameworks, inform more responsive practice, programming, and policy processes.

## 1 Introduction

The intersection of therapeutic, health, and well-being outcomes with leisure, recreation, and sport activities represents a significant area of study for understanding holistic approaches to improving the quality of life of persons with disabilities (1). Disability is a complex and multifaceted phenomenon that affects millions of children worldwide, including those in South Africa. The International Classification of Functioning, Disability and Health (ICF), defines “disability” as an umbrella term encompassing impairments, activity limitations, and participation restrictions, resulting from the interaction between a person's health condition and their contextual factors (2). In 2023 the United Nations Children's Fund (UNICEF) estimated there to be nearly 240 million children with disabilities in the world (3), with 207.4 million (12.5%) children aged 5–17 years and 236.4 million (10.1%) children aged 0–17 years that have moderate-to-severe disabilities (4, 5). Disability disproportionately affects vulnerable populations, and children from poorer households are at significantly higher risk of disability than other children (6). In 2011 South Africa had an estimated 2.9 million people living with a disability of which approximately 474,000 were children with some form of serious disability with many more having mild to moderate disabilities (7).

The health, wellness and development benefits of leisure, recreation, and sport activities represents a significant area of study for understanding holistic approaches to improving the quality of life for children with disabilities (8, 9). Although surf therapy programmes have shown evidence of physical, psychological, and psychosocial benefits for persons with disabilities (10) more needs to be known about the benefits of surf therapy for children with disability. Surf therapy is defined as “a physical activity intervention, combining surfing with structured activities promoting psychological, physical, and psychosocial well-being” (11). The first surf therapy peer reviewed publication appeared in 2010 (12). This small group intervention has been steadily growing, and in 2020 the first scoping review of both quantitative and qualitative evidence for its therapeutic outcomes emerged based on 29 studies over a ten year period that met the inclusion criteria for surf therapy (13). This scoping review highlighted many studies on surf therapy focusing on short-term outcomes, yet there is limited research on the long-term impact of programmes and outcomes for participants (13). The WHO recommends that children between the ages of 5 and 17 years participate in 60 min or more of moderate to vigorous intensity aerobic activity throughout the day (14). The aerobic activity requirements for children with disabilities are the same as those without a disability with due recognition that children with disability require purposefully designed leisure, recreation, and sporting activities to optimize the therapeutic benefits of their participation (15) The doctoral study of the lead author of the current article confirmed that structured engagement by children with disabilities in therapeutic surfing activities can produce observable and self-reported longitudinal improvements across physical, psychological, social, and emotional domains of functioning (16).

Surfing is a popular water sport that has been practiced for centuries around the world. It originated in Polynesia, where it was known as “he'e nalu” (wave sliding) and was an integral part of the culture (17). The sport was introduced to the Western world in the late 18th century by the famous explorer Captain James Cook, who

observed it during his travels in the Pacific. Since then, surfing has spread to all corners of the globe and has become a multibillion-dollar industry (18–20). There is no consensus on a single definition of surfing. Most definitions agree that surfing involves riding a wave on a board (21). Riding waves and carving boards is a product of various cultural, political, and environmental forces that play out in symbolic and corporeal forms that warrant discursive attention. Meanings go beyond the physical act of riding waves and include the cultural and social aspects of the sport.

Surfing is a physically demanding sport that requires a lot of muscle strength, balance, and cardiorespiratory endurance (22). The repetitive paddling motion helps to build upper body strength and improve core stability, which can also lead to better posture and balance (23–26). There is also increasing interest in the potential use of outdoor water environments, or blue space, such as in surfing, in the promotion of human health and well-being. A systematic review conducted by Britton, Kindermann, Domegan, and Carlin (27) of blue space interventions for health and well-being (known as blue care) highlighted thirty-three studies of blue space interventions specifically designed and structured with a therapeutic purpose. The review suggested that blue care can have direct benefit for mental health and psycho-social well-being (27).

The first special issue on “Surf Therapy Around the Globe” was released in the Global Journal of Community Psychology Practice, and included articles on emerging theory, research, and programme evaluation of eight surf therapy programmes across six countries (10). Of these eight programmes, three included children with disabilities (27–29) and one was located in South Africa, which focused on the evaluation of the process of adapting an existing surf therapy programme for neurotypical vulnerable youth to serve neurodiverse youth in the country (28). Sarkisian et al. (10) highlight that surf therapy programmes use four primary community psychology practice competencies, namely community inclusion and partnership, empowerment, mentorship, and health promotion. Marshall et al. (30) found these programmes have potential to provide a mechanism to fill the gap of unmet therapy needs and help overburdened medical systems in the UK (30). In South Africa, surf therapy programmes have been implemented to help children with disabilities improve their physical, emotional, and social well-being (31–33).

Surf therapy as a form of physical activity intervention is proving its feasibility and benefits for the physical fitness of children with disabilities. An eight-week surfing intervention study in the United States of America (USA) conducted by Armitano et al. (34) with 16 children with disabilities focusing on fitness testing showed improvements in participants' upper-body strength, grip strength in the upper extremities, range of motion, core strength, and cardiorespiratory endurance. A study conducted in the USA by Clapham et al. (35) investigated the effects of an eight-week surfing programme on the physical fitness of 71 children with a range of disabilities such as Autism spectrum disorder (ASD), Down Syndrome, global developmental delays, and cerebral palsy. The study focused on a comparison of the differences in overall fitness levels between the surf therapy group and an unstructured pool playgroup. The results demonstrated significant improvements in the surfing group in core strength, upper body strength, and cardiorespiratory endurance, while there were no significant differences in overall fitness levels between the surfing and unstructured pool playgroups. Body composition measurements on the surfing group demonstrated a significant reduction in total body fat percentage and fat free mass, and significant improvement in bone mineral density from pre- to post-surf therapy.

A qualitative study by Moore et al. (36), designed to understand parents' perceptions of their children's participation in a surf therapy programme in the USA, included ten parents and one caregiver. The results highlighted that parents perceive surf therapy to have positively impacted their child behaviourally, physically, and socially. Their study took place during a non-profit surf camp in California designed for children and young adults with ASD and aimed to promote awareness of the benefits that surf-focused recreational therapy programmes may have for children. The study found that children and youth were more socially interactive, engaged and more likely to be involved in other activities after participating in the surf therapy programme.

Britton, Kindermann, and Carlin (27) used a creative, participatory approach to evaluate an eight-week surf programme with twelve surfers as a surf therapy intervention for youth with ASD in the north-west of Ireland. A novel method of body mapping was used to evaluate the feelings and emotional well-being of young participants in the programme, which highlighted surfing as a beneficial psychosomatic experience. Findings showed how body mapping can be used to create a richer picture of the potential health and well-being outcomes from engaging with the sea. Children expressed feelings of happiness and freedom, while their parents reported that the children were more relaxed and confident.

Devine-Wright and Godfrey's (37) study with vulnerable youth in the UK demonstrated a sustained, positive impact of surfing on vulnerable youth's well-being over time. The research was conducted through The Wave Project, a UK-wide surf therapy charity organisation that provided vulnerable youth aged 8–21 years with an opportunity to surf once a week for six weeks. Findings showed that participants' well-being improved across seven locations in the south-west of England for up to three months after intervention. Participants showed transformation in their lives, including shifting from isolation to engagement with others through a combination of the surfing, volunteering, and mentoring offered in the programme.

Another study in the Netherlands by van Ewijk et al. (29) focused on the positive effects of surfing on psychological well-being from the parents' perspective for children with developmental difficulties between the ages of 8 and 18 years old. A positive effect of surfing was found on the quality of life of children participating. The study also highlighted a statistically significant increase in psychological well-being, social support, autonomy, and parental relations after three consecutive sessions. Van der Merwe and Yarrow (28), conducted a study in South Africa with children with ASD that explored the feasibility and unique benefits of an existing surf therapy programme for neurotypical children to see if it could be adapted to be more inclusive to meet the needs of neurodiverse children. The programme followed a structured 16-week programme that was delivered over a period of four months. Forty-five children between the ages of 13 and 17 participated, and a mixed-methods data collection approach was used, which included focus groups, surf mentor observations, and feedback on the beach. The study found that children with ASD experiences were predominantly positive and surf therapy had a positive effect on their overall well-being. It highlighted that the existing surf therapy programme can be offered as a meaningful, community-based mental health service to children with ASD, although not in the same format offered to neurotypical children as the original programme structure was not optimally inclusive and appropriate forneurodiverse children. The current article focuses on the narrative of one of five children with disabilities that participated in a surf therapy programme designed by the lead author as part of a PhD study in the Western Cape province of South Africa. An aim of the study was to explore the benefits of a surf therapy programme from the perspective of children themselves. Many studies on surf therapy focus on short-term outcomes, yet there is limited research on the long-term impact of programmes and outcomes for participants (13), which was addressed in this study.

## 2 Materials and methods

### 2.1 Research approach

A qualitative participatory research approach was used for this study to answer the research question: “How do children with disabilities experience their participation in a surf therapy programme in South Africa?”. The study concentrated on understanding how people make sense of the world by interpreting how they experience the events that they are participating in (38, 39). One aim of the study was to describe the effects on them during and after participating in a surf therapy programme, from the perspective of children with disabilities. The research design was a longitudinal exploratory case study of a social phenomenon ie. how children with disabilities experience their participation in a surf therapy programme (40). This form of case study was most appropriate for answering the research question as there were no predetermined benefits or outcomes (41). The research design had to situate surf therapy as a health promoting intervention, combining person-focused and environmentally based components of each child's narrative.

### 2.2 Theoretical and conceptual frameworks

The theoretical frameworks of this study were the Social-Ecological Model of Disability (42) and the Community-Based Rehabilitation (CBR) Guidelines of the World Health Organisation (WHO) to substantiate surf therapy as a strategy for disability inclusion (6) and the Bronfenbrenner's Bioecological Model of Human Development (43) to explain the multisystem benefits of surf therapy.

The Social-Ecological Model of Disability theorises disability as a product of multi-level interactions between individuals and their surrounding environments, shaped by both personal factors and broader social, cultural, and structural influences. The CBR Guidelines frame disability as a rights-based and socially constructed phenomenon, arising from the interaction between impairments and environmental barriers, and requiring inclusive, community-level responses to ensure participation and equity Bronfenbrenner's Bioecological Systems Theory (43) emphasizes the importance of understanding human development in the context of the complex and interconnected systems in which individuals live. It explores a child's development within the context of a system of relationships that form their environment (44) and the active role of the developing individual. To understand the effect of these proximal processes on development, one must focus on the person, context, and developmental outcome as these processes vary and affect people differently (45).The process of child development is not linear, and is in fact a complex system of relationships affected by multiple levels.

Each system can have bi-directional influences, which means that relationships have impact in both directions, both away from and towards the individual. Bronfenbrenner and Evans (45) also emphasise the importance of a positive relationship in overcoming the potential damage that is caused by a negative or ineffective environment. They state that even a constructive environment may not be sufficient to foster emotionally positive development without a warm and caring relationship. These five complex layers of environment are the microsystem (for example, immediate home, school, family, neighbourhood); mesosystem (for example, other homes, schools, families, neighbourhoods), exosystem (for example, mass media, parents workplaces, community services), macrosystem (for example, cultural values,beliefs, customs, laws) and chronosystem (for example global pandemics, educational milestones, relocation, family transitions) each of which influence a child's development. Conflict or change in one layer will create a ripple effect through to other layers. This theory focuses on a holistic approach to the interactions with a larger environment, and not only the child and their immediate surroundings.

### 2.3 Setting and participants

The surf therapy sessions were run at Muizenberg Beach, in Cape Town, South Africa. This location was chosen as it is suitable for its “learn to surf” wave, warmer waters of the False Bay coastline and suitable universal access facilities. The safety of the participants remained a priority of the research. The professional surf coaches had all received training in surf therapy and already worked extensively in a surf therapy programme. The overall surf therapy training includes a minimum of a level one first aid certification, level one Surfing South Africa or International Surfing Association accreditation (ISA Coaching, n.d.), and training in how to deliver surf therapy. As this intervention was an adapted surfing programme, there were various adaptations that needed to be made and the support team members needed to be trained in these. Adaptations included specialized surfboards with grip pads and handles, adjusted wetsuits and training in beach and water transfers. Participants were recruited using an information flyer at clinics and schools for children with disabilities.

The inclusion criteria for children were:

- Between 10 and 17 years old;
- Spoke either English or Afrikaans;
- A physical, sensory, or intellectual impairment.
- Never experienced ocean-based sports before or participated in a surf therapy programme.
- Comfortable going into the ocean
- Able to float appropriately and safely wearing a flotation device
- Willing to share about their experiences with another person.

Exclusion criteria:

- No children assessed at a level 3 or level 5 Surfer Ability Level were able toparticipate. This assessment was based on the Surfer Safety Level (SSL) and Surfer Ability Level (SAL) assessment provided by a paediatric neurodevelopmental physiotherapist trained in surf therapy.
- Children were asked to explain in their own words what the researcher had shared in the information sheet. If the child was not able to accurately express that they understood what the research would entail, they were not eligible to participate.
- Children that were non-verbal and children with severe cognitive impairments that may not be able to self-report or express accurately during interviews and during the initial assessment meeting.

### 2.4 Data gathering

Each child's voice was heard through the narration of their lived experiences before, during, and after participating in a surf therapy programme. The open-ended individual interviews were informed by narrative inquiry method, which records the perspectives of an individual on self-identified significant issues, revealing their lived experience of surf therapy. It reveals a deeper understanding of a situation, often giving a voice to marginalised populations (47). The children were asked to use their own words from their own experience. Their narrative story was guided by using open-ended questions during three interviews with each child.

### 2.5 Data analysis

Qualitative narrative data analysis involves the identification of patterns, concepts, meanings and themes in transcribed interview text (48). Narrative analysis was used because the research focus was interpretive, aiming to gain a phenomenological understanding of participant's experiences of surf therapy. The three interviews with each child were transcribed and combined into each child's single narrative (49).

### 2.6 Ethics

Ethical approval was obtained from the Human Research Ethics Committee in the Faculty of Health Sciences, at University of Cape Town (HREC#627/2020). Verbal and written proxy informed consent was obtained from parents/guardians, and informed assent from children, prior to participation. Age-appropriate, accessible information sheets were provided, and participants were given time to review and ask questions before signing. Consent/assent was voluntary, re-confirmed during follow-up interviews, and documented securely. Data was de-identified, pseudonyms used, and all materials stored confidentially on a password-protected system.

Ethical considerations with regards to participation were considered, including safety in the water. The potential risks were mitigated by the implementation of the comprehensive safety protocols in the surf therapy programme, contributing to avoiding any harm. Each team had up to 12 team members supporting each participant. Depending on the participants' profile, each lesson was run by a head coach, a head surf therapy volunteer, and a physiotherapist, with up to ten trained surf therapy volunteers in the water safety team. As the participants improved throughout the process, it became evident that they needed less support and fewer team members in the water. However, to ensure team cohesion, the same teams remained each week where possible. Life jackets and specialised adapted surfboards with additional floatation, grip, and handles were used.

The sessions took place in shallow water where the surfers were no more than about 1.25 m deep in the water. The surf coaches supported the participants where needed, and, where necessary, a second coach sat on the board providing seated support to the surfer. At the time of programme delivery, there was no formal training available in South Africa that provided a structured method for surf therapy. The best practice principles were developed and used based on an existing programme running for children with disabilities in Muizenberg.

As the research was conducted during the Corona Virus pandemnic, the researcher had to carefully consider the steps required to reduce and minimise risks related to COVID-19, drawing on the ethical principle of non-maleficence. The researcher had to take into consideration the status of the lockdown level in South Africa over the course of each month as the research continued, and review protocols to conduct the research and surf therapy under lockdown level 2. In addition they had to plan for any unforeseen changes that may have arisen during a time when change and uncertainty were frequent (50).

### 2.7 Findings: Rowan's story

Rowan (pseudonym) is one of five children that met all the inclusion criteria. His story is described in this article because it exemplifies the multisystem benefits of surf therapy, disability inclusion through CBR. Rowan began participating in the surf therapy research programme at the age of 14. He resided with his mother and two siblings in a brick built structure in Khayelitsha, one of Cape Town's most under-resourced and densely populated townships. He attended a government run primary school for children with disabilities, 22 kilometers (13.6 miles) from his home. Rowan was a quadruple amputee as a result of meningococcal disease contracted in early childhood. He spent large amounts of time undergoing surgery and in hospital growing up as a child. He used crutches and wore prosthetic lower limbs for mobility when he began the surf therapy research.

Rowan's narrative was recorded on May 2021, July 2021 and July 2022 as he shared his longitudinal experiences of the surf therapy programme. Follow-up interviews, one week after the surf therapy programme had been completed and one year later, helped to determine whether participants continued to experience benefits after the intervention ended. These follow-up interviews also provided valuable feedback for improving the surf therapy programme and tailoring it to participants' individual needs.

Rowans story is presented chronologically using headings that reflect narrative patterns, concepts and meanings. Interpretation of Rowan's narrative culminated in one main theme and three sub-themes that are presented in the next section of the article.

#### 2.7.1 May 2021

##### 2.7.1.1 About me

My mum, she works, she is a supermum, as everyone has been supermum for their children. My mum works at the hospital, she is a house mother there. And my sister, she also works in a private hospital. I have 2 siblings, ages 9 and 12 years. The one is smaller than me, the one is bigger than me, but I'm a bit older than her. They are at the same school. I'm a table tennis player, 3rd time Western Province bronze medalist. My first shot for the silver medal was in 2019 and I got a golden token in 2019 also for my class. My goal is to qualify for the Tokyo Opens this year at the Paralympics and play with the professional players, and get to play better and play for South Africa. I haven't chased my South African colours yet but I'm hoping I could this year. I also placed at Sas (South African National Championships). I got bronze and I'm happy with it. I only have 2 friends that have been true to me, Evan and Thabo. I met Evan at the Red Cross Hospital and have been friends for almost 13 years now. He is Mitchel's basketball player. Evan was 2 years old when he lost his leg in an accident. When he was small, he liked to play in the streets, he did not look around him when a bus ran over him. The doctors told him his leg will not be able to work so they decided to amputate his leg. Thabo had almost the same disease I had growing up. I enjoy school a lot. I enjoy being with my friends, learning in class and I like my teachers. I enjoy talking and I get to chat to my friends, laugh and ask for help in class. We like to play table tennis too. At home, I go to the sports field and I just play there. If I see there is a game going on, then I ask them to play, if it's rugby I play. If it's soccer, I stand at the poles and be the goalkeeper. There isn't anything I find difficult to do.

##### 2.7.1.2 Sport is my thing

I would love to join more sports. Rugby, I love everything about it. I like rugby because I have two favourite players and I want to play just like them. The one is a left wing Makazole Mapimpi and a right wing Cheslin Kolbe. I basically like the whole South African team, I like everyone there, they do their job, what they are asked to do, and what they are trained to do. It is what they were born to do, in the field. I play table tennis, I used to play with my friend but my friend passed away last year, it really made me sad when he passed on. I'm the most happy when my family and friends are around me and when they are positive. My wish is to become a SA table tennis player and I would also like to be better at school. I have been to the sea. I like going to any beach as long as I swim. I don't know much about surfing but I know that people are competing at the sea. I decided to try surfing because I like to be in the water, I can't swim well, but I try. I like to try fun stuff, I want to try surfing.

#### 2.7.2 July 2021

##### 2.7.2.1 I am born ready

My coach asked me if I am ready and I told him, “I'm born ready”. When I arrived the first day, I had so much energy. I went into the changeroom, my coach and my surf buddy helped me get changed. They duct taped the wetsuit legs and I walked out of the changeroom like a boxer going into a fight. Really amped. When we first walked to the water, I was a little nervous but I knew my team was in control. I was very excited to catch my first wave. I popped up standing and riding side on. I was the first to stand up. It was cool to be out in the water. Everyone being there by my side, they are “my team”, I was so blessed. I felt like I was getting used to it with my coach close by. We spent a lot of time practicing falling off the board too. I enjoyed it. On my last wave, I fell into the water and learnt what it is like to wipe out. The next surfing session was good, I learnt some new stuff that was amazing. My goal for the second session was to learn to paddle on my own. I learnt how to pop up and basically, I can pop up. One thing I learnt is that when I lean my shoulders to the back, the board is going to the front, and when I lean my shoulders to the front, my board is going to the back. I learnt how to pop off, jumping off the board. On my third session, I did well, I was so focused and determined to learn. I learnt to sway the board around, concentrating on my bodyweight and turning the board. Falling and turning around while I was floating and surfing on my own. I am happy about doing all those things successfully. When the session was over, I told my coach, “I just want to go on the board again”.

##### 2.7.2.2 When my sessions are done, I won't forget this day

The fourth session was very nice and I enjoyed it. It's one day I will remember. When my sessions are done, I won't forget this day. I was tired from a gym session I did that morning but when I went into the water, I had more energy. I wanted to see if I can get onto the board myself and I practiced that. What my coach taught me since last week is to move with my feet on the board. I never knew I could do that to sway my body around to catch some waves [adjust position on the board when riding a wave. I have learnt that I am strong in my core [torso] and arms, I can use that strength to sway and move my hips to turn. I started learning to turn and I enjoyed the smaller [narrower] board.

##### 2.7.2.3 I have learnt to trust in the team

The next session was very nice, I loved it. They taught me how to use my waist and core, open my body and close it again. I learnt a new thing, how to stop a board. Iwent back on the board and it literally stopped. I have learnt to trust in the team and I know when I fall my team will catch me. Surfing makes me feel relaxed. I forget about things when I do it with my friends. In the final session, the surfing was very good. I loved it. Especially the wave I caught on my own, I never knew I could do that. I was using everything I was taught over the last six weeks, but the stopping of the board, I just knew how to do it because I just felt crazy when I'm in the water. I figured that out on my own. I have learnt a lot about myself over this time. I learnt I can do it (surfing) because when I started I couldn't believe I could do it. I can't believe in six weeks I can turn the board. I learnt a lot about teamwork and appreciating relationships from this time with my team. I felt sad surfing is ending and I will miss my team all so much. I have learnt how to surf, I have learnt I can dream. It has totally been an honour to be here. I have been blessed and I am feeling grateful.

##### 2.7.2.4 If I can do it, I can do it for the rest of my life

Before I started surfing, I was thinking in my mind I can't do it [surfing] until I tried it and just being there was like beyond being able to speak in my wildest dreams. I couldn't believe I could surf in the ocean riding some waves. On my first session, I was like “If I can do it, I can do it for the rest of my life”. After my last session, I wanted extra weeks. I felt very down in my last session because I didn't know when I was going to see everyone again. I hope to see them all again. I learnt how to surf and I liked learning to surf on my own. I liked people guiding me. Paddling was a bit difficult as the board was wide in the beginning. I learnt how to steer the board, I never knew how to steer the board like that. My “pop ups” are much better since I started surfing. I learnt how to use the movements of my upper body. I learnt in my mind to stay focused. If a friend wanted to try surfing, I would basically tell them, “Ya, you can go, you can try surfing, surfing is real fun”. But I can't teach them because I am not at that level yet. They must try it if they want to.

##### 2.7.2.5 I would describe surfing as lovable, fun, exciting

I like everything about surfing like how people help me out in the ocean, catching some waves, but the most fun thing is, in the last session I finally caught my own wave and I never knew I could do that. I didn't like when the waves hit us and cracked over me, I just fell and people must help me up again. I would describe surfing as lovable, fun, exciting. My surfing experience has been fun, having everyone next to me and guiding me to do better, just learning how to surf.

#### 2.7.3 July 2022

##### 2.7.3.1 This year has been good and I made a lot more friends

I play rugby with friends in school and I've dubbed a single (song) last month I want to dedicate to my mom. That single is to thank my mom, to show how much I appreciate her. Because there's a lot of children that get murdered or their moms and dads have died and some people have lost their mothers during birth, and just a grateful feeling and that I still have a mom next to me. This year has been good and Imade a lot more friends, but I am a bit sad because I was separated from Evan. I managed to cope and I made a new friend but no one can replace Evan and the place in my heart. I still get to see him in the surf sessions. I have still carried on surfing.

##### 2.7.3.2 The journey of learning it's getting a lot easier since I am gaining knowledge

I'm still struggling a bit with paddling because my right arm is shorter than my left arm. So it is a bit of a disadvantage for me to paddle and some people are full bodied and they can paddle. The pop up is pretty easy because I know how to pop up and really taught myself to pop up. I like to learn new things. That's the thing. What I do is lay down flat and paddle and paddle. Once I had a wave take me, I can try to turn my board fully to the new place where the waves form up. I see the wave can take me with him and I turn my body, I look over my shoulder and turn to the right and that is how it goes. My favourite part about surfing is to learn new skills and to be in the water and surf. A year ago, I was just like a little boy running around the sand being stubborn and I had a lot of support next to me. When I fell, I couldn't pop up immediately and I was a learner. Just moving the board around wasn't that easy but now I have seen a lot of progress in myself turning the board, timing of the pop up, reading the wave and like there are no people behind me riding the wave, I now ride the wave on my own and I feel those changes are still with me.

##### 2.7.3.3 The moment I step my foot in the sand, I forget everything and start new on the beach

Academically, what I feel is when I come from school, I get more pressured and I also get anxieties and I stress more. I stress to the extent that when I write, I shake because I want to get my homework done on time. The moment I step my foot in the sand, my feet in the sand or the sea, I forget everything that happened in the past and I start new on the beach. When I am on the board, I suddenly focus on what is going to happen and the feeling I get is, it is just a great feeling of surfing. I think it's great and I think it's a pretty nice feeling. The changes I felt last year are still with me. A year ago, I was a bit panicky because it was my first time surfing and falling compared to now. Now I like to stress free—if I fall, it's like a sign of I'm trying and I know when I fall I just climb on the board again. So it is not like a train smash to fall because by making mistakes while learning and since I have the experience of falling off the board, it has taught me a lot.

##### 2.7.3.4 Balancing in surfing has helped out of the water too with my prosthetics

The experience of falling off the board has taught me balancing skills and to time myself when to go back and when to go forward and adjust my weight from one side to another. It has helped me out of the water too with my prosthetics, and with my balancing skills I managed to walk without my crutches, that is a big progress for me. And about my balance, it taught me a lot about balance because I left my crutches behind once and it felt like a panic, Evan told me to just relax. I remember my mom told me to relax. I was feeling the pressure of walking without my crutches and the day in school I was panicking and falling many times but then I learnt to walk without it. It's also when I walk, I think about the way I balance in surfing because I am on a board, I'm balancing so why can't I balance on prosthetics you see.

##### 2.7.3.5 My goal is to become a national surfing champion

The balance helped me surf in a competition, it was the provincial championships and I won. That was a taste of what competition is going to be like for me and I still managed to get first place. It taught me many things like to get more preparation and to get more practice. My highlight of surfing so far was when I managed to ride on a big wave but in the end I fell and that taught me a lot about it because when you make mistakes, no one is perfect and everyone makes mistakes. It is the only way I can learn and progress. Basically, I was learning a lot about learning and practicing a lot more because I wasn't used to the double up and I wasn't used to surfing on double ups. It was exciting because everyone just went mad when I rode that wave and in the end when I lost it, they still clapped hands and they told me I must practice more on balance, because they say in the Paralympics the waves are double the size of the waves at Muizenberg. My goal is to become a national champion and to become a Paralympic champion and to compete against guys that already have experience at nationals and SA's. That will just teach me a lot about the strategy of the game. I can describe my surfing experience so far as amazing, loving and learning.

### 2.8 Theme: an ambassador's journey of change

Rowan's longitudinal story reflects his positioning as an ambassador for disability inclusion. He described how learning to surf helped him to “*feel grateful,” “relax,” and “forget everything and start new*”. Across the interviews, his narrative showed how the experience supported a sense of possibility and belief in his own potential. He often referred to how he was supported by others and how this helped him to keep trying, even when it was difficult. As his confidence grew, he began to talk about encouraging others to try surfing, saying, “You can go, you can try surfing, surfing is real fun”. These reflections indicate a shift from focusing only on his own experience to sharing his story in a way that might benefit others. His ambassadorial role emerged through participating in surf therapy, with three sub themes capturing his emergent journey of development and change in all the systems of his life: 1. *Surfing has taught me to be more myself*'; 2. *Reshaping my worldview*; and 3. *Now I like to (am) stress free*.

#### 2.8.1 Sub-theme 1: surfing has taught me to be more myself

Rowan's journey highlights the role of surf therapy in promoting physical, mental, and emotional health. His participation led to notable physical improvements, including increased balance, posture, and mobility as well as mental, and emotional health improvements. During the second interview, (July 2021) Rowan described his experience in the first surf therapy lesson, when he accidentally left his crutches at a local store at the beach and felt panicked. He was always used to using crutches and did not walk without them. He spoke about his recognition that if he could balance on a surfboard riding a wave, he could balance on his prosthetics. The following week, he went to school without crutches and never used them again. His motivation, focus, and drive continued to grow and were observed during the second and third interviews.

#### 2.8.2 Sub-theme 2: reshaping my worldview

A sub-theme in Rowan's journey was the transformation of his perception of himself and the world around him. His participation in surf therapy challenged preconceived notions of disability and sport, allowing him to redefine what was possible. Through experiential learning, Rowan realized that difficult situations and physical challenges could be opportunities for growth rather than limitations. Rowan began to observe the world through his surf therapy experience, which changed his future outlook on life. In the second and third interviews (July 2021 & July 2022), Rowan spoke about the future outlook of his life. He spoke about his goal to become a national champion in South Africa and paralympic champion, competing on the global stage in para surfing. Rowan spoke about how he fell off a wave and the volunteers told him to keep practising because “*in the Paralympics, the waves are double the size of the waves at Muizenberg*”.

Rowan's journey continued. In June 2022, a year after his third interview, Rowan participated in the Western Province Provincial Championships and won his division, prone assist. Two months later, he placed second in the South African Championships and was selected for the 2022 South African National Team. Nineteen months after his first interview, Rowan's dream became a reality. In December 2022, on his 16th birthday, he competed at the World Para Surfing Championship at Pismo Beach, California, and placed 17th in the world. The following years, in April 2023 and May 2024 Rowan continued to build on his success in competitive parasurfing and won the 2023 and 2024 South African National Championships in his division.

#### 2.8.3 Sub-theme 3: now I like to (am) stress free

Rowan spoke about his experience as soothing and calming, which supported a reduction in overall anxiety. Learning to surf was part of the overall surf therapy programme that was delivered, which presented many opportunities for developing and mastering new skills. While the physical nature of the sporting activity was beneficial for Rowan's participation, he also developed skills in the social, psychological, and emotional domains through the activity of surfing.

## 3 Discussion

Rowan's journey of change, which began when he participated in surf therapy and eventually represented his country at the world championships, can be seen as an example of the potential benefits of surf therapy within Bronfenbrenner's (43) Bioecological Model of Human Development.

Rowan's narrative during and after surf therapy indicates that his disability was managed through the interaction between his strengths and limitations and the demands of the intersecting system environments within which he lived. This finding is similar to the findings of Moore et al. (36), Armitano et al. (34), and Clapham et al. (35) from studies conducted in the United States that involved children with disabilities as participants.

At the microsystem level, Rowan's participation in surf therapy provided him with opportunities for physical activity, skills development, and increased self-confidence. In adhering to the Social Ecological Model of Disability (42), the microsystem of the surf therapy programme created opportunity structures and offered supportive attitudes benefitting his health, shifted his worldview, and promoted his mastery of skills. The support and guidance from the surf instructors and individuals delivering the programme, along with the encouragement of bystanders, his mother and school, played a crucial role in nurturing his interest and talent. Through consistent participation, he gained confidence, improved balance and mobility, and developed coping strategies for managing anxiety. Surf therapy helped Rowan transition from reliance on crutches to walking independently with prosthetics, showing the tangible physical and psychosocial benefits of the programme at microsystem level. Learning to surf enabled him to “*dream*”. Sport had always been central to Rowan's identity, as he was a former Western Province table tennis player, and competitive sport was a recurring theme in his interviews. Following the initial six-week surf therapy programme, Rowan was one of five children with disabilities who went on to participate in parasurfing competitions. His pathway from surf therapy to competitive parasurfing illustrates how participation reshaped his worldview, expanded his aspirations, and transformed his personal sense of identity. Surf therapy created a space for Rowan to reimagine his future beyond limitation, fostering a mindset of resilience, self-worth, and ambition. He began to view challenges as opportunities, set new goals including becoming a national and international parasurfing competitor and achieved them. This shift reflects surf therapy's capacity to transform internalised narratives of disability into empowered identities (16).

Within the mesosystem, effective communication and collaboration between surf instructors, therapists, and his family were essential in identifying opportunities, securing resources, and providing continuity of support. As Rowan's surfing abilities progressed, these stakeholders worked together to support his continued growth and development. Other studies report on the significance of exosystem stakeholder collaboration to promote positive outcomes for children with disability in surf therapy (37, 51). Exosystem stakeholder collaboration is exemplified in multi-sectoral CBR Guidelines, positioning surf therapy as a scalable, community-based rehabilitation tool (6). Rowan's story shows that surf therapy can be effectively integrated into CBR Guidelines (6) for disability inclusive sport (37, 52, 53). His development was supported not just by the microsystem individualised surf therapy programme, but also by local community stakeholders surf instructors, schools, and sponsors illustrating how surf therapy can activate supportive ecosystems. Rowan's story of change validates surf therapy as a practical and scalable intervention for inclusion, especially in under-resourced contexts like South Africa. His experience highlights surf therapy's alignment with WHO's CBR Guidelines (6) and the Social Ecological Model of Disability (42), highlights the need for broader policy support and investment.

At the exosystem level, the broader community played a significant role. The surf therapy programme Rowan continued to attend is well integrated into a community that values acceptance and inclusivity. A local wetsuit manufacturer sponsored a customised wetsuit, and a surf school contributed additional surf lessons to support his training for competition surfing. The involvement of volunteers and mentors expanded his network of support beyond his immediate family, which was particularly significant during his transition from home life to dormitory accommodation (54).

At the macrosystem and chronosystem levels, Rowan's journey from surf therapy to representing South Africa at the World Para Surfing Championships highlights the importance of inclusive sports policies and disability rights. His progression demonstrates how enabling environments, inclusive opportunities, and community investment can create pathways for children with disabilities to thrive (43). It emphasises the importance of policy support and investment to scale surf therapy as a community-based inclusive development strategy.

### 3.1 Implications for practice and policy

Rowan's narrative illutrates the significance of future development of surf therapy within the South African context and beyond. The programme adaptations made across the longitudinal data gathering have already shaped aspects of provincial planning, illustrating how surf therapy can serve as a model for inclusive, community-based recreational programming. Rowan's narrative contributes to surf therapy scholarship by identifying the mechanisms of change underlying participant improvement not merely reporting outcomes.

Unlike most surf therapy studies that end immediately after programme completion, Rowan's narrative provides rare longitudinal evidence, demonstrating sustained benefits one year later. His narrative also expands the global evidence base by offering insight from a Global South, low resource, educational disability context, where surf therapy remains under-researched.

Rowan's expereince shows how surf therapy operates across multiple ecological systems individual, family, peer, school, and community highlighting improvements in family relationships, communication, and social engagement. By linking surf therapy with broader ecological and relational systems, the case story offers a deeper understanding of how inclusive coastal programming can support educators, therapists, and policymakers in designing more accessible, community rooted interventions for children with disabilities.

## 4 Conclusion

An inclusive surf therapy programme benefits the health and development of children with disabilities through reshaping their worldview, enabling the mastery of new life skills and enhancing their self-confidence beyond the surf board into multiple spheres and systems of their lives. Rowan's journey from surf therapy participant to international parasurfing competitor demonstrates the transformative potential of inclusive sports programmes for children with disabilities. His story illustrates how opportunities created within supportive ecological systems, from family and community stakeholders to policy frameworks, can foster physical, psychological, and social development. The visibility of his achievements challenges societal perceptions of disability, inspires other children with disabilities, and highlights the need for sustained investment in inclusive sport. At a broader level, Rowan's success demonstrates how surf therapy and similar community-based interventions can inform policy, strengthen accessibility, and expand opportunities for participation, ultimately advancing disability inclusion in sport and society (55–58).

## Data Availability

The raw data supporting the conclusions of this article will be made available by the authors, without undue reservation.
